# A Novel Approach to Bacterial Expression and Purification of Myristoylated Forms of Neuronal Calcium Sensor Proteins

**DOI:** 10.3390/biom10071025

**Published:** 2020-07-10

**Authors:** Vasiliy I. Vladimirov, Viktoriia E. Baksheeva, Irina V. Mikhailova, Ramis G. Ismailov, Ekaterina A. Litus, Natalia K. Tikhomirova, Aliya A. Nazipova, Sergei E. Permyakov, Evgeni Yu. Zernii, Dmitry V. Zinchenko

**Affiliations:** 1 Laboratory of pharmacokinetics, Department of Biological Testing, Branch of Shemyakin and Ovchinnikov Institute of Bioorganic Chemistry of the Russian Academy of Sciences in Puschino, Pushchino, 142290 Moscow Region, Russia; vladimirov@bibch.ru (V.I.V.); rinarowing@mail.ru (I.V.M.); 2 Department of Cell Signaling, Belozersky Institute of Physico-Chemical Biology, Lomonosov Moscow State University, 119992 Moscow, Russia; vbaksheeva@belozersky.msu.ru (V.E.B.); tikhomir@belozersky.msu.ru (N.K.T.); zerni@belozersky.msu.ru (E.Y.Z.); 3 Faculty of BioMedPharmTechnological, Pushchino State Institute of Natural Sciences, Pushchino, 142290 Moscow Region, Russia; 4 Laboratory of New Methods in Biology, Institute for Biological Instrumentation of the Russian Academy of Sciences, Federal Research Center “Pushchino Scientific Center for Biological Research of the Russian Academy of Sciences”, Pushchino, 142290 Moscow Region, Russia; ismailov_ramis@mail.ru (R.G.I.); ealitus@gmail.com (E.A.L.); alija-alex@rambler.ru (A.A.N.); permyakov.s@gmail.com (S.E.P.); 5 Institute of Molecular Medicine, Sechenov First Moscow State Medical University, 119991 Moscow, Russia

**Keywords:** recoverin, guanylate cyclase activator protein 1 (GCAP1), guanylate cyclase activator protein 2 (GCAP2), neuronal calcium sensor 1 (NCS-1), neurocalcin δ (NCALD), myristoylation, N-myristoyl transferase

## Abstract

N-terminal myristoylation is a common co-and post-translational modification of numerous eukaryotic and viral proteins, which affects their interaction with lipids and partner proteins, thereby modulating various cellular processes. Among those are neuronal calcium sensor (NCS) proteins, mediating transduction of calcium signals in a wide range of regulatory cascades, including reception, neurotransmission, neuronal growth and survival. The details of NCSs functioning are of special interest due to their involvement in the progression of ophthalmological and neurodegenerative diseases and their role in cancer. The well-established procedures for preparation of native-like myristoylated forms of recombinant NCSs via their bacterial co-expression with N-myristoyl transferase from Saccharomyces cerevisiae often yield a mixture of the myristoylated and non-myristoylated forms. Here, we report a novel approach to preparation of several NCSs, including recoverin, GCAP1, GCAP2, neurocalcin δ and NCS-1, ensuring their nearly complete N-myristoylation. The optimized bacterial expression and myristoylation of the NCSs is followed by a set of procedures for separation of their myristoylated and non-myristoylated forms using a combination of hydrophobic interaction chromatography steps. We demonstrate that the refolded and further purified myristoylated NCS-1 maintains its Ca^2+^-binding ability and stability of tertiary structure. The developed approach is generally suited for preparation of other myristoylated proteins.

## 1. Introduction

Neuronal calcium sensors (NCSs) are EF-hand Ca^2+^-binding proteins responsible for transduction of calcium signals into a wide range of neuronal responses by regulating activity of effector enzymes and other target proteins (for review, see [1]). Due to these properties, NCSs are involved in a number of processes crucial for neuronal function, including reception, neurotransmission, synaptic plasticity, neuronal growth and survival [2,3,4,5,6,7]. Growing evidence indicates that NCSs are involved in the pathogenesis of neurological and neurodegenerative disorders as well as different types of cancer [8,9,10,11]. In addition, NCSs were recognized as redox-sensitive proteins, which may underlie their pathophysiological activity in oxidative stress-related disorders [12,13,14].

NCSs protein family includes 14 members characterized by different distribution in the nervous system. Thus, recoverin and guanylate cyclase-activating proteins (GCAPs) are mostly specific for photoreceptor cells of the retina; visinin-like proteins (VILIPs) are expressed throughout the cerebral cortex, the midbrain and the cerebellum, as well as in retinal and olfactory neurons [15,16,17,18]; neurocalcin δ (NCALD) is found in most neurons of the central nervous system, in the retina and the adrenal gland [19,20]; Kv channel-interacting proteins (KChIPs) are characterized by varied localization throughout the brain, and are also present in the heart muscle and male reproductive system [21]; neuronal calcium sensor-1 (NCS-1) is expressed ubiquitously in the central and peripheral nervous systems, including the innervation of the gastrointestinal tract and endocrine glands, in mast cells of the blood, as well as in smooth muscle and in the developing heart [15,22,23,24,25,26]. NCSs possess common domain architecture consisting of N-terminal and C-terminal domains, each containing two structurally coupled EF-hand motifs. Among these motifs, only EF2 and EF3 (recoverin), EF3 and EF4 (KChIP1) or EF2, EF3 and EF4 (other KChIPs, GCAPs, VILIPs, NCS-1) are capable of binding calcium or magnesium, whereas EF1 is always non-functional due to the absence of certain amino acids responsible for metal ion coordination. Due to specific structural features, such as different organization of Ca^2+^-binding loops and the presence of specific regulatory elements (for instance, C-terminal segment [27,28,29]), each NCS binds calcium within its own narrow range of the cation concentration, which allows these proteins to respond to different Ca^2+^-signals and play the role of Ca^2+^-buffer in cells [30]. Alongside calcium and magnesium, NCSs are capable of binding zinc, which is suggested to be important for their normal and pathological activity [31,32].

N-terminal myristoylation is an indispensable modification of the majority of NCS proteins [33]. It is believed to occur co-translationally (after cleavage of the initiator methionine by methionyl aminopeptidase), but may also represent post-translational modification, accomplished after proteolytic cleavage of the N-terminal glycine residue [34]. Basically, myristoyl group is responsible for targeting NCS proteins to different membranes, thereby governing their intracellular localization and compartmentalization with multiple binding partners. Most of the NCSs bind to phospholipid membranes via the so-called Ca^2+^-myristoyl switch mechanism, which is best characterized in case of recoverin [35]. In the absence of Ca^2+^, myristoyl group is buried inside a hydrophobic pocket in the recoverin molecule. Sequential binding of two calcium ions to EF3 and EF2 of the protein leads to extrusion of the myristoyl group, thereby anchoring the protein in the lipid bilayer. In recoverin, the functioning of the Ca^2+^-myristoyl switch and subsequent membrane binding are affected by a number of different factors, such as proper structure and conformation of its unique N-terminal and C-terminal segments, electrostatic contacts between the protein and the membrane, phosphatidylserine content and potential of the membrane as well as the presence of zinc and redox state of the medium [12,31,36,37,38,39]. NCALD [40], VILIP1 [41] and VILIP3 [42] possess a similar Ca^2+^-myristoyl switch mechanism, whereas NCS-1 has its myristoyl group permanently exposed in the presence of membranes [28,43,44,45] and its membrane localization is predominantly governed by the interaction of the protein with specific phospholipids in the bilayer [46]. In contrast to the other NCSs, in GCAP1 the myristoyl group is not involved in membrane binding. Instead, it participates in a Ca^2+^-dependent activator-to-inhibitor transition of the protein with respect to its targets, retinal guanylate cyslases, through the so-called Ca^2+^-myristoyl tug mechanism [47,48]. In general, the role of myristoyl group in NCSs is not limited to membrane anchoring. Thus, it was found to contribute to overall structural stability of these proteins, thereby affecting their critical functional features, such as calcium sensitivity and target recognition [48,49,50,51,52]. It is suggested that in Ca^2+^-free NCSs intramolecular interactions between the myristoyl group and the binding pocket residues shape these homologous proteins into different structures, which therefore expose different sets of hydrophobic residues in response to calcium binding. Apparently, this mechanism provides for the specificity of NCSs association with different physiological targets, thereby diversifying their intracellular activities [53]. 

Given the critical importance of the myristoyl group for proper folding and functioning of NCSs, it is clear that any in vitro study involving these proteins requires preparations of their myristoylated forms. While the production of intrinsically myristoylated NCSs in animal cell lines is characterized by low yields, their generation in bacteria presents a number of technical problems. Since N-myristoyl transferases are absent in *Escherichia coli*, myristoylation of NCSs is normally achieved by their co-expression with the respective enzyme from *Saccharomyces cerevisiae* (NMT1p) [33]. Unfortunately, NMT1p exhibits different specificity with respect to substrate sequence as compared to the mammalian enzyme, resulting in unstable and sometimes low acylation of NCS proteins in bacteria [54]. In some cases, this problem can be solved by point mutations, which makes myristoylation signal more appropriate for NMT1p. For instance, mutation D6S was demonstrated to enhance myristoylation of GCAP1 [55]. Nevertheless, although myristoylation level of bacteria-expressed recoverin, VILIP1, VILIP3 and neurocalcin δ can reach 80–95% of the total protein [56,57,58,59], in case of GCAPs this value is about 50–80% [60], whereas for NCS-1, it does not exceed 60% [43,61]. On the other hand, the separation of myristoylated and non-myristoylated forms of NCSs is a complicated task as currently it is only achieved by reverse phase high-performance liquid chromatography (HPLC) that may result in irreversible denaturation of the target proteins and is not suitable for obtaining their preparative quantities. An attempt to solve this problem was made in case of NCS-1 by providing it with C-terminal 6×His-tag, which increased solubility of the protein and allowed for chromatographic separation of its myristoylated and non-myristoylated forms [62]. Yet, any approach involving introduction of 6×His-tag in NCSs is questionable, since modification of their N-terminus is impossible due to myristoylation, whereas modification of C-terminus might affect the function of these proteins [27,28,29,63,64]. 

With this in mind, in this study we aimed at developing a novel approach for obtaining native-like myristoylated forms of various NCSs, including recoverin, GCAP1, GCAP2, NCALD and NCS-1. To this end, we analyzed and optimized their expression and myristoylation in bacteria and adopted a simple method for separation of their myristoylated and non-myristoylated forms by a combination of hydrophobic chromatography steps performed under non-denaturing conditions. In addition, we examined feasibility of insoluble expression, refolding and purification procedures for obtaining myristoylated NCS-1 with intact structural and functional properties. We propose that general approach suggested in this study may be considered upon developing novel efficient techniques for expression and purification of different proteins bearing myristoyl group.

## 2. Materials and Methods 

### 2.1. Materials

Chromatographic resins Phenyl Sepharose, Butyl Sepharose and CNBr-activated Sepharose 4B were from GE Lifesciences (Marlborough, MA, USA); Butyl Toyopearl was from Tosoh Bioscience (Griesheim, Germany). The SDS-PAGE molecular weight standards and bicinchoninic acid (BCA) protein assay kit were from Thermo Fisher Scientific (Rockford, IL, USA). Other reagents were obtained from Sigma-Aldrich (St. Louis, MO, USA), Merck (Darmstadt, Germany), Fluka (Buchs, Switzerland), PanReac AppliChem ITW Reagents (Darmstadt, Germany), Serva (Heidelberg, Germany) and Amresco (Solon, OH, USA) and were at least analytical grade. All buffers and other solutions were prepared using ultrapure water.

### 2.2. Antibodies

Rabbit polyclonal antibodies against recoverin, NCS-1, GCAP1 and GCAP2 were prepared previously [28,65,66]. Polyclonal anti-NCALD antibodies were produced by rabbit immunization. The generated antibodies were purified from serum using a resin with immobilized antigen [67]. To obtain the resin, recombinant NCALD was conjugated to CNBr-activated Sepharose 4B (GE Lifesciences) according to the manufacturer’s instructions. The antibody titers were monitored by Western blotting. 

### 2.3. Genetic Constructs

Plasmid vectors for expression of recoverin (in pET-11d) and NCS-1 (in pET-22b) genes were obtained in previous studies [28,68]. The genetic constructs encoding GCAP1 and GCAP2 (in pET-11a) were kindly provided by Prof. Karl-Wilhelm Koch (University of Oldenburg, Oldenburg, Germany) [55]. Bovine NCALD gene was obtained by reverse transcription and PCR amplification using total mRNA purified from *Bos taurus* brain tissue. The resulting amplicon was subcloned to pET-22b(+) expression vector for Escherichia coli using endonucleases sites HindIII and NdeI by the standard cloning techniques. 

### 2.4. Expression Protocol

Expression of NCS proteins was performed in *Escherichia coli* strains BL-21(DE-3) Star, or BL-21(DE-3) Codon Plus RIL. The cells transformed with NCS-expressing vectors were cultivated on Petri dishes with agarized LB medium (10 g/L NaCl, 10 g/L triptone, 5 g/L yeast extract, 1.5% agar) containing 50 μg/mL ampicillin. To obtain myristoylated proteins, the cells were co-transformed with plasmid pBB131 bearing NMT1p gene [69] and cultivated in the same medium in the presence of 50 μg/mL ampicillin and 10 μg/mL of kanamycin. The resulting strains were stored by freezing freshly grown culture (OD_595_ = 1.0) in 15% glycerol at –70 °C. 

For expression of NCS proteins, 50 mL of LB medium (10 g/L NaCl, 10 g/L triptone, 5 g/L yeast extract) containing 0.1% glucose was inoculated with a single colony from a fresh dish prepared by overnight cultivation of cells from a frozen glycerol stock. The cells were cultivated to OD_595_ = 1.0 at 37 °C with continuous shaking (250 rpm) for 6–8 h and kept overnight at +4 °C. The next morning, 5 mL of the cell suspension was added to 500 mL of glucose-free pH-buffered LB medium (10 g/L NaCl, 10 g/L triptone, 5 g/L yeast extract, 50 mM Na_2_HPO_4_, pH 8.0) containing the respective antibiotics. In the case of obtaining myristoylated NCSs, the cells were grown to OD_595_ = 1.0 and myristic acid (50 mg/mL stock in 96% ethanol) was added to final concentration of 20 mg/L. At OD_595_ = 1.5–2, IPTG was added to final concentration of 0.5 mM and the shaking was continued for additional 4–5 h (250 rpm, 37 °C).

### 2.5. Extraction of Recoverin, NCALD and NCS-1 as Soluble Proteins

After the expression, the cells expressing recoverin, NCALD or NCS-1 were centrifuged at 6000× *g* for 10 min, the supernatant was discarded, and the cells were resuspended in lysis buffer containing 50 mM Tris-HCl (pH 7.5), 5 mM MgCl_2_, 1 mM EDTA, 0.1 mM PMSF and 3 mM DTT (25–50 mL for 500 mL of the cell culture). The lysates were frozen at -20 °C, thawed at room temperature, and kept on ice with lysozyme (25–50 µg/mL) for 20–40 min with stirring. The resulting suspension was centrifuged for 20 min at 12,000× *g*. If necessary, the pellet was subjected to another freeze/thaw cycles to extract additional 10–20% of the protein. 

### 2.6. Extraction from Inclusion Bodies as Insoluble Protein and Renaturation of NCS-1, GCAP1 and GCAP2

After the extraction of soluble NCS-1, the cell pellets containing residual insoluble protein were resuspended in lysis buffer (2–5 mL for 500 mL of cell culture) and the suspension was added, drop by drop, to dissolution buffer (25 mL for 500 mL of cell culture) containing 20 mM Tris-HCl (pH 7.5), 100 mM NaCl, 1 mM EGTA, 2 mM MgCl_2_, 6 M urea and 1 mM DTT. GCAPs-containing pellets were treated similarly except for dissolution buffer contained 7M guanidine hydrochloride instead of urea. The mixture was kept dissolving overnight at 4 °C with gentle stirring and then dialyzed against 10 mM Tris-HCl-buffer (pH 7.5), 100 mM NaCl, 1 mM EGTA, 2 mM MgCl_2_, 1 mM DTT (1000–10000 w/w) two times for 3 h with the buffer exchange. The resulting fraction was centrifuged for 30 min at 20,000× *g*, and the supernatant was used for chromatographic purification.

### 2.7. Purification of Myristoylated Recoverin, NCALD and NCS-1 by Hydrophobic Chromatography

For purification of recoverin and NCALD, calcium chloride was added dropwise to the cellular extracts to reach the final concentration of 3 mM (on ice). The resulting fraction was centrifuged for 30 min at 20,000× *g*, and the supernatant was loaded onto Phenyl Sepharose column (GE Lifesciences), equilibrated with 20 mM Tris-HCl-buffer (pH 7.5), 2 mM CaCl_2_, 2 mM MgCl_2_ and 1 mM DTT. The elution was performed using (1) 20 mM Tris-HCl-buffer (pH 7.5), 2 mM MgCl_2_, 1 mM DTT and 1 mM EGTA and (2) deionized water. The bulk of the myristoylated proteins was eluted in step (1).

For purification of NCS-1, the respective cellular extract was mixed with 1 M NaCl (to reduce protein aggregation) and calcium chloride was added as described above. After the centrifugation (30 min at 20,000× *g*), the mixture was loaded onto Phenyl Sepharose column, equilibrated with 20 mM Tris-HCl-buffer (pH 7.5), 2 mM CaCl_2_, 2 mM MgCl_2_, 1 mM DTT. The elution was performed in three steps using the following conditions: (1) 20 mM Tris-HCl-buffer (pH 7.5), 1 mM EGTA, 2 mM MgCl_2_ 1 mM DTT and 200 mM NaCl; (2) 20 mM Tris-HCl (pH 7.5), 1 mM EGTA, 2 mM MgCl_2_ and 1 mM DTT; (3) deionized water. The bulk of the myristoylated protein was eluted in steps (2) and (3).

To accomplish final separation of myristoylated from of NCS-1, the fractions obtained in the steps (2) and/or (3) were mixed with NaCl to reach the final concentration of 1 M and loaded onto Butyl Sepharose column, equilibrated with 20 mM Tris-HCl-buffer (pH 7.5), 1 mM EGTA, 2 mM MgCl_2_ and 1 mM DTT, containing 1M NaCl. The target protein was eluted by reducing NaCl concentration in the same buffer using linear or step gradient. If necessary, myristoylated recoverin was purified from the EGTA fraction by the same procedure. The approximate gradient conditions required for elution of myristoylated NCS-1 and recoverin are indicated in Table 2. If degree of NCSs myristoylation was still below 90%, the chromatography on Butyl Sepharose column was repeated.

### 2.8. Purification of Myristoylated GCAPs by Hydrophobic Chromatography

The fractions containing renaturated GCAP1 and GCAP2 were mixed on ice with NaCl to reach its final concentration of 1 M, and the resulting mixture was loaded onto Toyopearl Butyl column equilibrated with 20 mM Tris-HCl-buffer (pH 7.5), 1 mM EGTA, 2 mM MgCl_2_, 1 mM DTT and 1M NaCl. The elution was performed in three steps using the following conditions: (1) 20 mM Tris-HCl-buffer (pH 7.5), 1 mM EGTA, 2 mM MgCl_2_ 1 mM DTT and 200 mM NaCl; (2) 20 mM Tris-HCl-buffer (pH 7.5), 1 mM EGTA, 2 mM MgCl_2_ 1 mM DTT and 100 mM NaCl; (3) 20 mM Tris-HCl (pH 7.5), 1 mM EGTA, 2 mM MgCl_2_ and 1 mM DTT. The bulk of the myristoylated proteins was eluted in step (2) and (3).

If necessary, the final separation of myristoylated forms of GCAPs was achieved using Butyl Sepharose chromatography. To this end, the fractions obtained in the step (2) and (3) were mixed with NaCl to reach the final concentration of 1 M and loaded onto Butyl Sepharose column, equilibrated with 20 mM Tris-HCl-buffer (pH 7.5), 1 mM EGTA, 2 mM MgCl_2_ and 1 mM DTT, containing 1M NaCl. The target protein was eluted by reducing NaCl concentration in the same buffer using linear or step gradient. The approximate gradient conditions required for elution of myristoylated NCS-1 and recoverin are indicated in Table 2.

### 2.9. HPLC Detection of The Myristoylated Protein Forms

The degree of NCSs myristoylation was determined by analytical HPLC (Waters Breeze) using 3.9 × 150 mm Luna C18 reversed-phase column (Phenomenex, Torrance, CA, USA)) in acetonitrile-water system [27]. First, 20 µL samples containing ~0.1–1 mg/mL of the target proteins were mixed with 50 µL of 100% acetonitrile, incubated with shaking for 30 min and centrifugated 8000× *g*. Then, 20 µL of supernatant were loaded onto the column equilibrated with water solution containing 2% acetonitrile and 0.1% TFA. The separation of myristoylated and non-myristoylated forms of recoverin, GCAP1 and GCAP2 was accomplished using linear gradient from 0 to 95% acetonitrile in water solution containing 0.1% TFA in the course of 30 min. In the case of NCS-1, linear gradient from 30 to 60% acetonitrile in the same solution was employed. The protein detection was performed by measuring absorbance at 280 and 215 nm. The purified non-myristoylated NCS proteins (obtained in the absence of NMT1p) were used as control. 

### 2.10. Mass Spectrometry Analysis 

Prior to analysis, recoverin and NCS-1 were dialyzed against ultrapure distilled water and mixed with acetonitrile (50%) and formic acid (5 mM). The resulting samples (100 μL) containing 5 μg/mL proteins were applied by direct injection. The mass spectrum was recorded in the positive mode from 600 to 1400 m/z (scanning speed 125 amu/s, detector voltage 1.5 kV) and a constant nitrogen flow of 1.5 l/min using Shimadzu LCMS-2010EV single quadrupole mass spectrometer (Shimadzu Co., Kyoto, Japan) with an electrospray ionization. The flow rate was 40 μL/min. The device was pre-calibrated using horse heart myoglobin. 

### 2.11. Fluorescence Measurements

Fluorescence emission spectra of NCS-1, recoverin and GCAP2 (4.8 μM) were measured at different temperatures using a Cary Eclipse spectrofluorimeter (Varian Inc., Mulgrave Victoria, Australia) equipped with a Peltier-controlled cell holder, mainly as previously described [28,29,32]. The measurements were carried out in 10 mM HEPES-KOH, 100 mM KCl, pH 7.6 buffer, in the presence of 1 mM CaCl_2_ or 1 mM EDTA. Excitation wavelength was 280 nm. All spectra were corrected for spectral sensitivity of the instrument and described by a log-normal function [70] using LogNormal software (IBI RAS, Pushchino, Russia).

### 2.12. Analytical Procedures 

SDS-PAGE and Western blotting was performed using the standard procedures [71,72]. Concentration of proteins was measured spectrophotometrically using previously calculated extinction coefficients at 280 nm of 24,075 M^−1^cm^−1^ (recoverin) [73], 21,430 M^−1^cm^−1^ (NCS-1) [28], 28,378 M^−1^cm^−1^ (GCAP1), 37,512 M^−1^cm^−1^ (GCAP2) [74] and 20,000 M^−1^cm^−1^ (NCALD) [75]. In some cases, protein concentration was verified using BCA protein assay. 

## 3. Results

### 3.1. Analysis of Myristoylation and Solubility of NCS Proteins Expressed in Bacterial Cells

**Figure 1:**
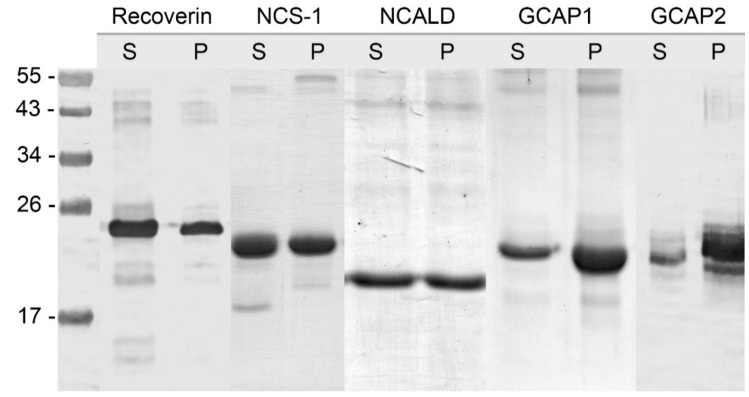
Solubility of neuronal calcium sensor (NCS) proteins expressed in bacteria. Western blotting of recoverin, NCS-1, neurocalcin δ (NCALD), guanylate cyclase activator protein 1 (GCAP1) and GCAP2 in soluble (‘S‘) and pellet (‘P‘) fractions obtained upon lysis of bacterial cells followed by centrifugation. The numbers in left-hand column indicate the molecular masses of protein standards in kDa.

Given that previous studies report contradictory information regarding solubility and myristoylation levels of recombinant NCSs [43,55,59,61,62,76], we firstly reproduced and verified these data. Five NCSs, namely recoverin, NCALD, GCAP1, GCAP2 and NCS-1, were expressed in bacteria according to the standard protocol and distribution of their myristoylated and non-myristoylated forms between soluble and insoluble fractions was studied. According to Western blotting data, NCSs significantly differed in terms of solubility in bacteria (Figure 1). 

**Figure 2:**
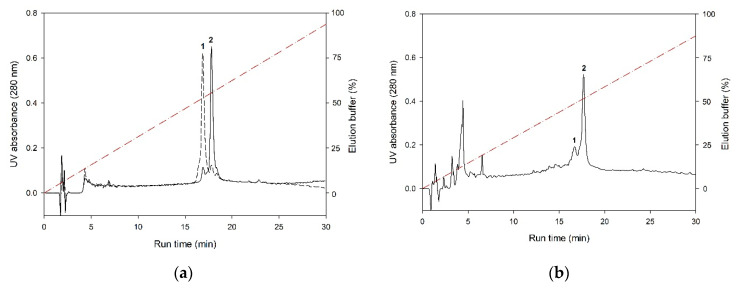

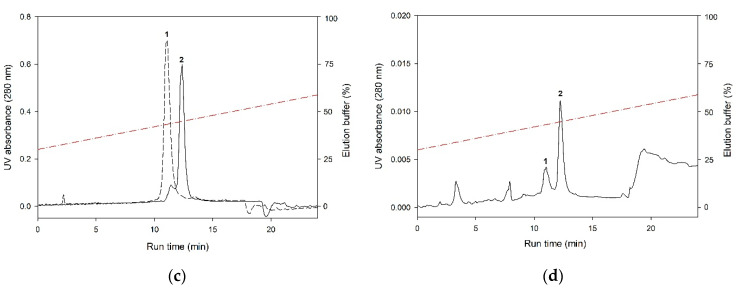
Distribution of recombinant recoverin and NCS-1 between soluble and insoluble fractions obtained upon lysis of bacterial cells expressing these proteins. Representative profiles of reverse phase HPLC of soluble fraction of bacterial cells expressing recoverin (**b**) and insoluble fraction of bacterial cells expressing NCS-1 (**d**). For comparison, HPLC profiles of the purified recoverin (**a**) and NCS-1 (**c**) are demonstrated. Non-myristoylated and myristoylated forms of both proteins are denoted as 1 and 2, respectively. UV absorbance at 280 nm is showed as a function of run time. Dashed red lines represent 0–95% (**a**,**b**) or 30–60% (**c**,**d**) acetonitrile gradient in water solution containing 0.1% TFA.

Thus, recoverin was expressed mostly as a soluble protein and GCAPs were detected predominantly in the membrane fraction, whereas NCS-1 and NCALD were almost equally distributed between the supernatant and the pellet. To assess myristoylation levels of the expressed NCSs, their soluble and insoluble fractions were additionally examined by analytical HPLC. Prior to the analysis, the insoluble fractions were treated with guanidine hydrochloride. The chromatographic separation of myristoylated and non-myristoylated forms of NCSs based on differences in their hydrophobicity was performed using high-resolution reversed-phase column (C18). In our experiments, the best fractionation was achieved using water-acetonitrile gradient (0–90% over the course of 30 min) in the presence of 0.1% TFA. Under these conditions, recoverin (Figure 2A), GCAP1, GCAP2 and NCALD exhibited similar elution profiles, where non-myristoylated form was eluted first. In case of NCS-1, efficient peak resolution was achieved employing more slopping gradient (30–90% over the course of 30 min) (Figure 2C). According to the HPLC data, the myristoylated forms of recoverin (Figure 2B) and NCALD were predominantly present in the soluble fractions (Table 1). Thus, we confirmed that these proteins can be purified directly from the cellular extracts. Meanwhile, substantial fractions of myristoylated NCS-1 (Figure 2D) and especially GCAPs were found in the insoluble fractions (Table 1). 

This does not necessarily point to their accumulation in the inclusion bodies, as both proteins are known to exhibit high membrane affinity regardless of the presence of calcium [43,77]. Nevertheless, their purification required additional treatment of the pellets, such as using sonication, or otherwise these proteins could be extracted with chaotropic agents, i.e., in denatured form. Since NCSs are recognized as redox-sensitive proteins [12,13,14], the sonication procedure was considered undesirable as it could cause thiol oxidation and alterations in structure and function of these proteins. We concluded that purification of GCAPs and NCS-1 may involve their renaturation after extraction from bacterial pellets as insoluble proteins. In case of NCS-1, the fraction containing renatured protein could be mixed with its soluble extracts, thereby increasing the total yield of the myristoylated form.

### 3.2. Optimization of Myristoylation of NCS-1 in Bacterial Cells

In our experiments, NCS-1 exhibited generally low level of myristoylation: about 50–60% of the total expressed protein lacked this modification. Therefore, we next modified the cultivation procedure in attempt to optimize expression and myristoylation of NCS-1 in bacteria. In particular, we varied conditions of the cell growth, such as pH, temperature and duration, as well as time points for induction and addition of myristic acid and analyzed the resulting products by means of analytical HPLC. The most pronounced benefit was observed upon alkalization of the cell growth medium: the incubation of the cells in LB medium buffered at pH 8.0 did not negatively affect NCS-1 expression, but increased its myristoylation up to 60–70%. It should be noted that the doubling time for bacterial cells cultured at pH 8.0 increased about 1.5-fold as compared to their cultivation at pH 7.0 (standard LB medium).

### 3.3. Primary Purification of Myristoylated Recoverin, NCALD and NCS-1: Phenyl Sepharose Chromatography

**Figure 3:**
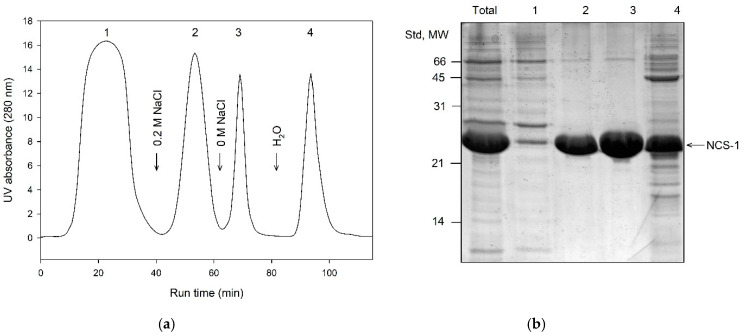
Primary purification of NCS-1 using Ca^2+^-dependent hydrophobic interaction chromatography on Phenyl Sepharose. (**a**) Representative chromatographic profile. The numbers 1–4 denote fractions obtained under the following conditions: 2 mM CaCl_2_, 2 mM MgCl_2_, 1 M NaCl and pH 8.0 (flow throw; **1**); 2 mM EGTA, 2 mM MgCl_2_, 200 mM NaCl and pH 8.0 (**2**); 2 mM EGTA, 2 mM MgCl_2_ and pH 8.0 (**3**), deionized H_2_O (**4**). (**b**) SDS-PAGE of the fractions **1**–**4**. The first track contains a total protein extract loaded onto the column. The positions of molecular weight standards (in kDa) are denoted in the left-hand column.

The well-accepted primary step for purification of recoverin, NCALD and NCS-1 is Ca^2+^/affinity chromatography on Phenyl Sepharose [14,19,33]. The procedure is based on ability of the NCS proteins to reversibly expose their hydrophobic target-recognizing site (hydrophobic pocket), in response to binding/chelation of the calcium ions [35]. Considering that myristoylation can differently affect overall hydrophobicity of NCSs, we analyzed distribution of their myristoylated and non-myristoylated forms in Phenyl Sepharose chromatography fractions. Protein extracts containing recoverin, NCALD or NCS-1 (obtained from soluble fraction or from insoluble fraction after refolding) were loaded onto the column equilibrated with low ionic strength/high calcium buffer and eluted with (1) the same buffer containing calcium chelator EGTA and (2) deionized water. The resulting fractions were analyzed for the myristoylation rate of the contained protein using analytical HPLC (Table 1). In case of recoverin, the treatment of the column with EGTA-containing buffer resulted in release of the relatively pure protein with degree of myristoylation of 90–95%, whereas for NCALD, this procedure yielded protein myristoylated by 97%. In contrast, in case of NCS-1, the respective fraction contained NCS-1 myristoylated only by 5–25%, whereas the rest of the modified protein (40–60% of myristoylation) was found in the water fraction. Given that NCS-1 was structurally unstable in water and the respective fraction contained multiple contaminations, the procedure of Phenyl Sepharose chromatography of NCS-1 was modified as follows (Figure 3). The protein was loaded onto the column in high ionic strength/high calcium, washed with high ionic strength/high magnesium and eluted by gradual reduction of the ionic strength in the presence of EGTA and Mg^2+^ (Figure 3). Under these conditions, the most of the non-myristoylated NCS-1 was eluted at 200 mM NaCl, whereas the treatment with salt-free Mg^2+^/EGTA-containing buffer yielded a large fraction of the protein with myristoylation level of 40–60%. Overall, we demonstrated that a combination of Ca^2+^/affinity and hydrophobic interaction chromatography on Phenyl Sepharose can be used as first step for separation of myristoylated and non-myristoylated forms of recoverin, NCALD and NCS-1 yielding fractions enriched in their myristoylated forms.

### 3.4. Primary Purification of Myristoylated GCAP1 and GCAP2: Toyopearl Butyl Chromatography

**Figure 4:**
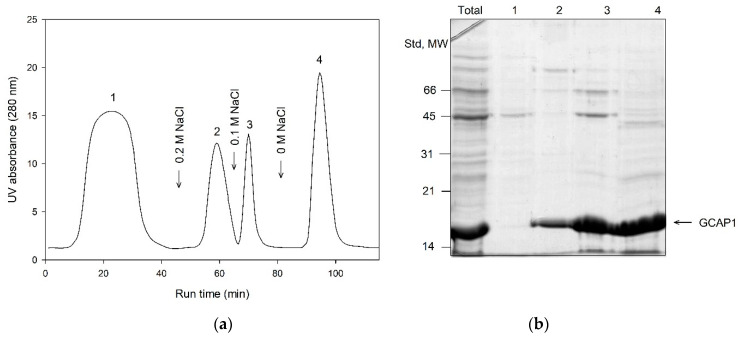
Primary purification of GCAP1 using hydrophobic interaction chromatography on Toyopearl Butyl. (**a**) Representative chromatographic profile. The numbers 1–4 denote fractions obtained under the following conditions: 1 M NaCl, 2 mM MgCl_2_ and pH 8.0 (flow throw; **1**); 200 mM NaCl, 2 mM MgCl_2_ and pH 8.0 (**2**); 100 mM NaCl, 2 mM MgCl_2_ and pH 8.0 (**3**), 2 mM MgCl_2_ and pH 8.0 (**4**). (**b**) SDS-PAGE of the fractions **1**–**4**. The first track contains total protein extract loaded onto the column. The positions of molecular weight standards (in kDa) are denoted in the left-hand column.

In contrast to the other NCSs, myristoylated GCAPs were mainly expressed in bacteria as insoluble proteins. Nevertheless, both proteins exhibited incomplete myristoylation in bacterial cells (approximately 80%, Table 1), which still necessitated separation of their myristoylated forms. Our preliminary experiments revealed that this separation can be accomplished after refolding and it occurred more efficiently when GCAPs were free from contamination of bacterial proteins. To accomplish such primary purification, we employed, for the first time, hydrophobic interaction chromatography on Toyopearl Butyl (Figure 4). Both GCAPs were bound to this resin under high ionic strength conditions (1 M NaCl) in EGTA-containing buffer and eluted by reverse salt gradient in the same buffer at 200 mM NaCl, yielding relatively pure proteins. Myristoylation level of the resulting products remained at 75–80%, indicating that myristoylation forms of GCAPs were completely preserved during this purification step. It should be emphasized that the described protocol was valid only when GCAPs maintained their Ca^2+^-free conformation during the chromatographic process. Notably, most of the commercially available sodium chloride reagents contain admixtures of calcium chloride, yielding approximately 1–100 µM Ca^2+^ in 1 M NaCl solution. Thus, the presence of background concentration of calcium chelator, such as 250 µM EGTA, is strictly required for proper purification of GCAPs (as well as NCS-1, see above), as they exhibit submicromolar affinities to calcium [32,78,79,80]. 

### 3.5. Final Separation of Myristoylated Forms of NCS Proteins: Butyl Sepharose Chromatography

**Figure 5:**
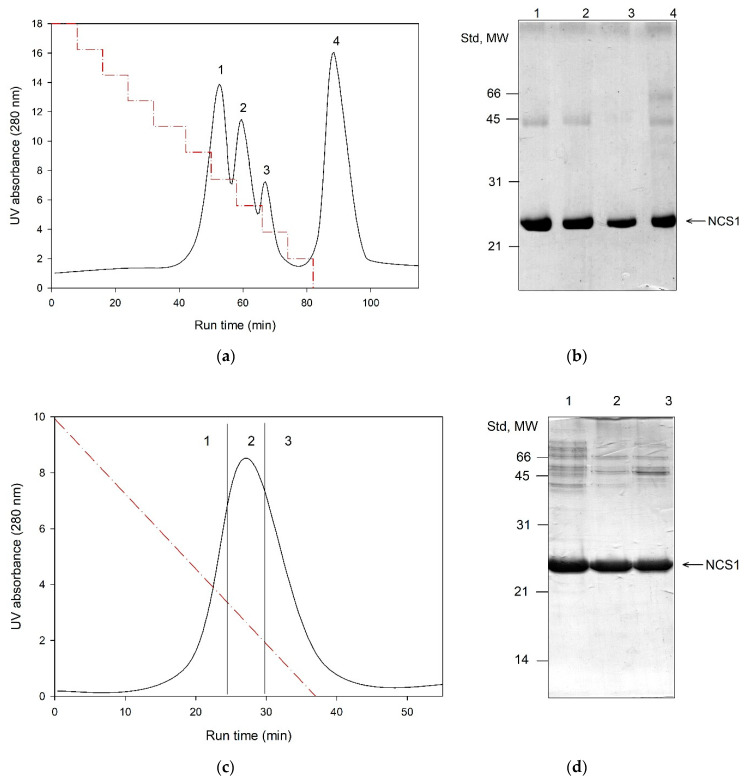
Final separation of myristoylated form of NCS-1 using hydrophobic interaction chromatography on Butyl Sepharose. (**a**) Representative chromatographic profiles obtained using linear gradient of NaCl. The numbers 1–4 denote fractions obtained under the following conditions: 800–600 mM NaCl, 2 mM MgCl_2_ and pH 8.0 (**1**); 600–300 mM NaCl, 2 mM MgCl_2_ and pH 8.0 (**2**), 300–100 mM NaCl, 2 mM MgCl_2_ and pH 8.0 (**3**); 100–0 mM NaCl, 2 mM MgCl_2_ and pH 8.0 (**4**). (**b**) Representative chromatographic profiles obtained using step gradient of NaCl. The numbers 5–8 denote fractions obtained under the following conditions: 400 mM NaCl, 2 mM MgCl_2_ and pH 8.0 (**5**); 300 mM NaCl, 2 mM MgCl_2_ and pH 8.0 (**6**), 200 mM NaCl, 2 mM MgCl_2_ and pH 8.0 (**7**); 2 mM MgCl_2_ and pH 8.0 (**8**). (**c,d**) SDS-PAGE of the fractions **1**–**4** (**c**) and **5**–**8** (**d**). The positions of molecular weight standards (in kDa) are denoted in the left-hand columns. The content of myristoylated NCS-1 contained in each fraction is indicated in Table 2.

The primary steps of purification described above yielded relatively pure NCSs enriched in myristoylated forms. To complete the purification procedure, we next searched for universally valid method for total removing of the admixture of the non-myristoylated forms from the NCSs preparations. Analysis of different hydrophobic resins revealed that Butyl Sepharose was the most suitable for this purpose. Indeed, all NCSs bound to this resin at high ionic strength (1 M NaCl) in EGTA/Mg^2+^-containing buffer and their non-myristoylated and myristoylated forms were fractionated by reducing salt concentration (for NCALD, this step is normally not required due to high degree of myristoylation of the protein obtained after Phenyl Sepharose chromatography). The best separation was achieved by employing step gradient rather than linear gradient of NaCl concentration (Figure 5, Table 1). The approximate gradient conditions favoring elution of non-myristoylated and myristoylated forms of recoverin, NCS-1, GCAP1 and GCAP2 are summarized in Table 2. Importantly, the exact NaCl concentration required for the elution of myristoylated form of each of the NCSs depended on the degree of its myristoylation in the loading fraction. Therefore, if possible, this parameter should be controlled (i.e., by analytical HPLC) during the purification. 

It should be noted that the introduction of Butyl Sepharose chromatography not only allowed separation of myristoylated forms of NCS proteins, but also lead to their more efficient purification from other admixtures. For instance, this step resulted in separation of NCS-1 from its proteolyzed fragment (18 kDa), commonly found in different preparations of the protein [81]. In the case of recoverin purification, Butyl Sepharose step efficiently substituted anion-exchange chromatography. MonoQ resin is normally used for ion-exchange chromatography in the final step of recoverin purification procedure, yielding protein preparation free of admixtures absorbing at 260 nm (nucleic acids).

### 3.6. Characterization of The Purified Myristoylated NCS Proteins 

To verify myristoylation level and structural integrity of NCS proteins obtained using the described new protocols, the following additional studies were performed. Firstly, LC/ESI-MS analysis was employed to check molecular weights of these proteins. For the analysis, we selected two NCSs, NCS-1 and recoverin, both obtained from soluble bacterial extracts by a combination of Phenyl Sepharose (primary purification) and Butyl Sepharose (final separation) chromatography. In this set of experiments, we also compared myristoylation of two variants of NCS-1, namely obtained from soluble and insoluble/refolding fractions. Preparations of non-myristoylated NCS-1 and recoverin expressed in bacteria in the absence of NMT1p and purified using the standard methods [82] were used as a control. It was found that average molecular weights of non-myristoylated and myristoylated (both soluble and refolded) forms of NCS-1 equaled to 21,746 and 21,957 Da, respectively. In turn, molecular weights of the corresponding recoverin forms were detected as 23,201 and 23,411 Da (Table 1). Considering the calculated molecular weights of NCS-1 (21,747.53 Da) and recoverin (23,202.22 Da) polypeptides lacking N-terminal methionine and the average mass change due to myristoylation (210 Da), the myristoylated forms of both proteins were identical to their native variants. Given that myristoylation involves N-terminal glycine [34], these full-size proteins represented NCS-1 and recoverin with myristoyl group attached to G2. It should be emphasized that, according to mass spectrometry, the obtained preparations of myristoylated NCS-1 and recoverin were free of non-myristoylated proteins. 

**Figure 6:**
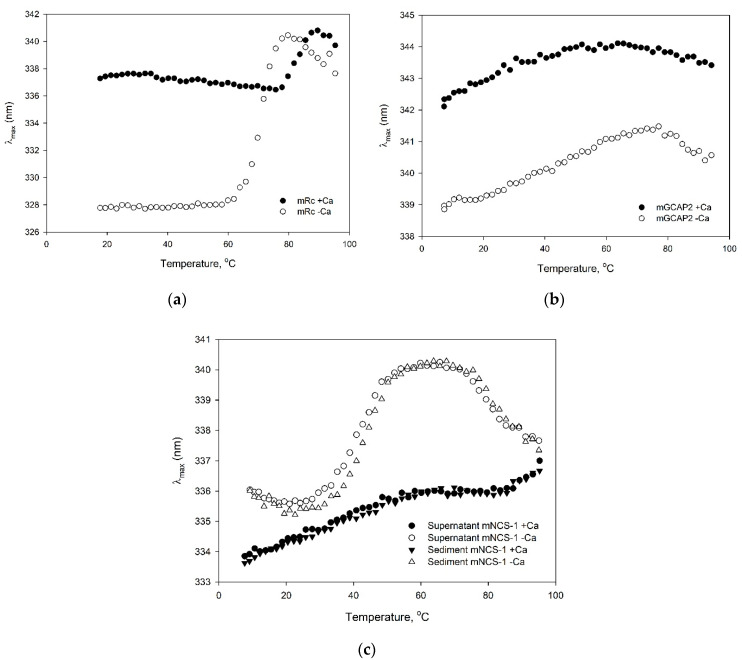
Thermal denaturation of apo- (1 mM EGTA, open circles) and Ca^2+^-bound (1 mM CaCl_2_, solid circles) forms of the purified myristoylated NCS proteins (4.8 μM), monitored by their tryptophan fluorescence (λ_max_, fluorescence spectrum maximum position): recoverin (**a**), GCAP2 (**b**) and NCS-1 purified from either soluble or insoluble fractions (**c**). Excitation wavelength was 280 nm.

Secondly, we analyzed structural stability and Ca^2+^-binding ability of the purified NCS samples by examination of thermal stabilities of their Ca^2+^-free and Ca^2+^-bound states. Temperature dependencies of tryptophan fluorescence spectrum maximum position for myristoylated recoverin, GCAP2 and NCS-1, purified from either soluble or insoluble fractions, are shown in Figure 6. The myristoylated recoverin sample demonstrated a Ca^2+^-induced red shift of the fluorescence spectrum (consistent with exposure of the emitting Trp residue(s)), and a cooperative thermal transition (Table 1), which shifted towards higher temperatures upon Ca^2+^ binding (Figure 6A), in accord with previous observations [14,27,29,39,64]. Similarly, Ca^2+^ binding induces red shift in GCAP2 fluorescence spectrum, but fluorescence technique did not reveal cooperative thermal transitions in this case (Figure 6B) [29]. The NCS-1 samples exhibited similar thermal denaturation profiles (Figure 6C) characteristic for this protein [28,32]: Ca^2+^ binding is accompanied by a blue shift of the fluorescence spectrum (indicates burial of the emitting Trp residue(s)) and a pronounced stabilization of its tertiary structure (Table 1). Hence, the refolding of myristoylated NCS-1 did not affect stability of its tertiary structure and Ca^2+^-binding ability. Overall, the myristoylated NCS samples prepared in the present study preserved their native tertiary structure and Ca^2+^-binding ability. 

## 4. Discussion

**Figure 7:**
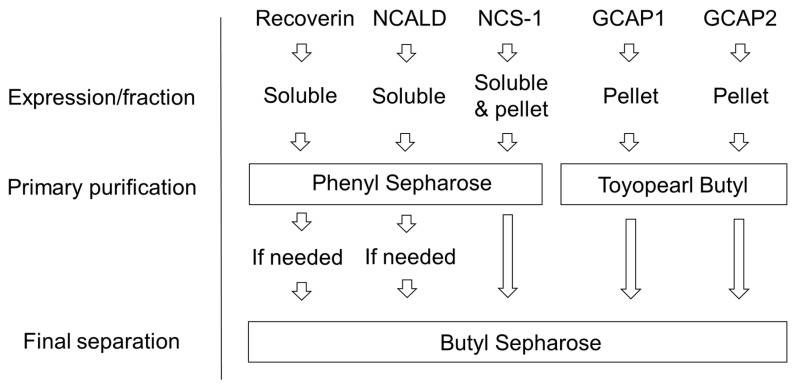
A summary of the purification steps for myristoylated forms of NCS proteins.

In this study, we report a novel approach for purification of nearly completely myristoylated forms of NCS proteins belonging to four out of five subgroups of the family, namely recoverin (subgroup ‘recoverins’), NCALD (subgroup ‘visinin-like proteins’), GCAP1/GCAP2 (subgroup ‘GCAPs’) and NCS-1 (subgroup ‘frequenins’) [83]. In particular, we optimized expression and myristoylation of these proteins in bacteria and developed a set of new procedures for separation of their myristoylated and non-myristoylated forms by a combination of hydrophobic interaction chromatography steps (Figure 7). Indeed, these forms differ in their hydrophobic properties because the presence of a myristoyl group not only makes its own contribution as a non-polar moiety (such as under the denaturing conditions of reverse phase HPLC), but significantly perturbs non-polar sites of the folded protein [53]. Notably, hydrophobic properties of NCSs display Ca^2+^-dependence and vary significantly among these proteins. Functionally, it can be observed from the different behavior of NCSs with respect to membrane binding and target recognition. In the current work, we employed all these features as the basis for developing optimized purification approach, the hallmark of which is that the separation of myristoylated and non-myristoylated forms of NCSs is conducted under non-denaturing conditions, thereby fully maintaining their structure. The method for production of myristoylated NCSs can be represented by a simple protocol consisting of four major steps, namely expression in bacterial cells, extraction, primary purification and final separation (Figure 2). All these steps differed among the NCS proteins as they were fine tuned in such a way as to maximally preserve and accumulate the respective structurally distinct myristoylated forms.

Generally, our approach improved purification of all myristoylated NCSs as compared to previously published methods. However, in case of each NCS, the benefit of these modifications was different. Thus, in case of recoverin and NCALD our modifications only moderately contributed the overall efficiency of the purification. The distinctive features of these proteins is that (1) they are well myristoylated in bacteria, (2) their myristoylated forms are extracted as soluble proteins and (3) both their non-myristoylated and myristoylated forms interact with Phenyl Sepharose in the presence of calcium, but the myristoylated form is eluted separately from (prior to) the non-myristoylated form upon chelating the cation. Such behavior can be explained using the example of recoverin based on ANS/Bis-ANS fluorescence probing of its non-polar properties [84]. Thus, (1) surface hydrophobicity of recoverin is significantly increased upon binding calcium and (2) non-myristoylated Ca^2+^-free recoverin is more hydrophobic than myristoylated Ca^2+^-free recoverin. Consistently, in our experiments the bulk of non-myristoylated Ca^2+^-free recoverin was eluted from Phenyl Sepharose only upon its washing with deionized water. In the aggregate, these features make it possible to purify recoverin (and NCALD) containing 90–95% of myristoylated form in a single step (Ca^2+^/affinity chromatography on Phenyl Sepharose) yielding relatively pure products. Yet, it is often necessary to have high-purity myristoylated proteins, which can be obtained by our Butyl Sepharose chromatographic procedure giving almost 100%-myristoylated form.

In contrast to recoverin and NCALD, GCAPs do not possess a functional Ca^2+^-myristoyl switch, and their myristoyl group is sequestered inside the protein globule, which makes the structure of their Ca^2+^-free and Ca^2+^-bound conformers less different (at least in case of GCAP1) [50,79]. Consistently, these proteins do not change or even decrease their surface hydrophobicity upon calcium binding [29,85] and Ca^2+^/affinity chromatography on Phenyl Sepharose is not applicable for their purification. Nevertheless, both GCAPs are generally very hydrophobic [29,85], and therefore, can be purified using Ca^2+^-independent hydrophobic interaction chromatography. This approach was employed in previous studies for GCAP1 purification: after the gel filtration step the protein was purified on Butyl Sepharose column without using salt gradient [86]. Alternatively, the gel filtration step was complemented by anion-exchange chromatography [60]. In our opinion, these variants have one significant limitation, namely, they do not separate myristoylated and non-myristoylated forms of GCAPs and the content of the myristoylated form is not being controlled during the purification. Indeed, in our experiments GCAPs were characterized by initially lower level of myristoylation in cells than recoverin and NCALD, but the bulk of their myristoylated form accumulated in insoluble fraction and was thereby separated. Yet, the content of myristoylated GCAPs in this fraction is still approximately 80% and, according to our observations, their level can be less in bacterial cells, which necessitates separation of the acylated form. In the current study, we resolved this problem by using hydrophobic Butyl Sepharose chromatography and fine-tuned ionic strength gradient. Since fractionation of the myristoylated and non-myristoylated forms after refolding occurred more efficiently when they were free from contamination of bacterial proteins, we also introduced a primary purification step employing Toyopearl Butyl. The use of this variant of hydrophobic chromatography instead of conventional gel filtration was justified by the fact that the latter has a number of limitations, such as low amount of the loaded sample per chromatography round, as well as dilution and loss of the target protein. Overall, our procedure allowed obtaining preparative amounts of GCAPs with degree of myristoylation of 96–98%.

However, the most significant achievement of our approach can be recognized in case of isolation of myristoylated NCS-1, which was initially the most problematic in this regard. Previous studies reported several procedures for the production of this protein, but neither of them yielded its highly myristoylated native-like form. In early works, NCS-1 has been purified using a two-step method originally developed for recoverin and NCALD [82], which included Ca^2+^/affinity chromatography on Phenyl Sepharose or similar resins as a single step [61] or with subsequent purification using ionic exchange chromatography or gel filtration [52,87]. However, the myristoylation level of the protein obtained with this method did not exceed 58–60% [43,61]. In general, Ca^2+^-dependent hydrophobic chromatography is hardly suitable for isolation of myristoylated NCS-1. Indeed, apo-from of this protein is unstable and, according to ANS/Bis-ANS probing, even more hydrophobic than its Ca^2+^-bound form. In turn, Mg^2+^-bound/Ca^2+^-free NCS-1 is less hydrophobic than Ca^2+^-bound NCS-1, but the difference between non-polar properties of these forms [28,32] is not sufficient to ensure complete elution of the former from Phenyl Sepharose upon calcium chelation. More recently, an alternative method of NCS-1 purification was suggested, which involved using N-terminally myristoylated NCS-1 expressed with C-terminal 6×His-tag [62]. Remarkably, the authors managed to separate its myristoylated and non-myristoylated forms using Ni-NTA resin and demonstrated that the tagged protein retains its overall structure and general ability to bind Ca^2+^or phospholipids. Yet, multiple studies conducted by our [27,28,29,63,64] and other [88,89] laboratories indicate that the structure and conformation of C-terminal segment of NCSs are critical for ensuring their precise Ca^2+^-sensitivity and specific regulatory activity. Thus, the introduction of 6×His-tag in C-terminus of NCS-1 may affect position of its flexible C-terminal segment and, consequently, specific functional properties of the protein.

In the current study, we propose a novel approach for obtaining untagged NCS-1 characterized by high degree of myristoylation (>95%). In particular, we made the following observations. (1) The level of NCS-1 myristoylation in bacterial cells is relatively low as compared to other NCSs, but it can be enhanced by increasing pH of the growth medium from 7 to 8. (2) Although some of the myristoylated NCS-1 is transferred into the bacterial extract, the substantial part of this form remains in insoluble fraction, but it can be extracted using chaotropic agent and purified after refolding with maintaining structure and Ca^2+^-binding properties of the protein. (3) Myristoylated NCS-1 demonstrates partially Ca^2+^-irreversible interaction with Phenyl Sepharose, but it can be separated from non-myristoylated form by loading the sample on the resin under high calcium conditions and elution by gradual reduction of the ionic strength in the presence of calcium chelator and magnesium. (4) Myristoylated and non-myristoylated forms of NCS-1 can be efficiently separated in the presence of Mg^2+^ by means of Butyl Sepharose chromatography using step gradient of ionic strength. 

Generally, the factors underlying the initially low degree of NCS-1 myristoylation in bacterial cells and the revealed alkalization-induced boost of the myristoylation remain scarce. NCS-1 contains a somewhat suboptimal NMT1p-binding site with a charged residue (lysine) in position 3, which was suggested to be unfavorable for yeast NMT binding, as compared to the mammalian enzyme [54,90]. However, virtually the same site is present, for instance, in NCALD, which exhibits a much higher degree of myristoylation (Table 1). Although alkalization of the bacterial medium induces an abrupt jump of intracellular pH, the latter undergoes relatively fast restoration [91]. Yet, it is suggested that bacteria may respond to alkaline stress by moderate increase in cytoplasmic pH and activation of enzymes with optimum activity at high pH levels [92]. Among these enzymes can be NMT1p, which doubles its activity with an increase in pH from 7 to 8 [93].

The revealed novel behavior of NCS-1 in hydrophobic chromatography is in agreement with previous evaluations of non-polar properties of its forms using ANS/Bis-ANS [52,78]. Thus, non-myristoylated apo-NCS-1 exhibited more pronounced hydrophobicity than myristoylated apo-NCS-1 resembling recoverin in this respect [84]. Meanwhile, in the presence of Mg^2+^ this trend became reverted as both forms demonstrated decrease in non-polar properties, but in case of non-myristoylated NCS-1 this decrease was much more pronounced. Taken together, these effects account for efficient resolution of NCS-1 forms during both types of hydrophobic interaction chromatography demonstrated in this study.

## 5. Conclusions

In this study, we suggested a novel approach for obtaining various NCSs, including recoverin, GCAP1, GCAP2, neurocalcin δ and NCS-1, ensuring their nearly complete N-myristoylation. In particular, we optimized bacterial expression and myristoylation of the NCSs and developed a set of procedures for separation of their myristoylated and non-myristoylated forms using a combination of hydrophobic interaction chromatography steps. The obtained preparations of NCS proteins with native-like modification are required for conducting proper structural and functional studies in vitro. We can suggest that general structural principles underlying the proposed approach may be common for a wide range of proteins bearing myristoyl group [94], and therefore, can be applicable for their efficient isolation.

## Figures and Tables

**Table 1:** Quantitative characteristics of NCS expression and purification. Primary purification steps: Phenyl Sepharose for NCS-1, NCALD and recoverin; Butyl Toyopearl for GCAPs. Final separation steps: Butyl Sepharose for NCS-1, GCAP1 and GCAP2.

Protein		Expression		Primary Purification	Final Separation	Molecular Weight, kDa ± SD *	Mid-Transition Temperature, °C **
		Supernatant	Pellet				
Recoverin	Content, mg	110	30	70	65	23411.0 ± 1.7	66.5
	Purity, %	70	30	96	98		
	Degree of myristoylation, %	80–90	90	90–95	99		
NCALD	Content, mg	90	25	60	50	-	-
	Purity, %	65	35	97	99		
	Degree of myristoylation, %	90	90	97	99		
GCAP1	Content, mg	25	50	15	10	-	-
	Purity, %	50	65	85	95		
	Degree of myristoylation, %	0	80	75–80	98		
GCAP2	Content, mg	15	35	12	10	-	no cooperative transition
	Purity, %	0	60	85	94		
	Degree of myristoylation, %	0	80	75–80	96		
NCS-1	Content, mg	80	60	42	15	21957.0 ± 1.4	43.6
	Purity, %	70	50	80	97		
	Degree of myristoylation, %	20	80	40–60	98		

* According to LC/ESI-MS data. ** According to monitoring of temperature dependencies of tryptophan fluorescence spectrum maximum position of the apo-form.

**Table 2:** Approximate content of myristoylated NCSs in the fractions from Butyl Sepharose chromatography upon use of step gradient of NaCl.

Protein	Degree of Myristoylation, %					
	Loading Fraction	400 mM NaCl	300 mM NaCl	200 mM NaCl	100 mM NaCl	0 mM NaCl
Recoverin	96	-	-	10	50	99
NCALD	97	-	-	10	50	99
NCS-1	50	10	30	70	85	96
GCAP-1	75	-	2	15	85	98
GCAP-2	80	-	5	20	90	98

## Abbreviations

NCS	neuronal calcium sensor;
NCS-1	neuronal calcium sensor-1;
NMT1p	yeast N-myristoyltransferase;
HPLC	high performance liquid chromatography;
FPLC	fast protein liquid chromatography;
GCAP1	guanylate activating protein 1;
GCAP2	guanylate activating protein 2;
NCALD	neurocalcin δ;
DTT	1,4-dithiothreitol;
EGTA	ethylene glycol-bis-N,N,N′,N′-tetraacetic acid;
Bis-ANS	4,4’-Dianilino-1,1’-Binaphthyl-5,5’- disulfonic acid, dipotassium salt;
LC/ESI-MS	liquid chromatography electrospray ionization tandem mass spectrometric;
BCA	bicinchoninic acid assay;
TFA	trifluoroacetic acid;
ANS	8-anilino-1-naphthalenesulfonic acid;
LB	lysogeny broth;
SDS PAGE	sodium dodecyl sulfate-polyacrylamide gel electrophoresis.
